# Eosinophil count at intensive care unit admission was not predictor of hospital mortality: results of a case control study

**DOI:** 10.1186/s40560-015-0093-4

**Published:** 2015-06-06

**Authors:** Emmanuel Jesús Escobar-Valdivia, Julio Edgardo González-Aguirre, Eunice Rebeca Carrillo-Cisneros, Karla Carolina Guerra-Leza, Roberto Mercado-Longoría

**Affiliations:** Department of Internal Medicine, “Dr. José E. González” University Hospital, Nuevo León Autonomous University, Av. Francisco I. Madero1500, Suburb: Mitras Centro, Monterrey, NL 64460 Mexico; Department of Pulmonary and Critical Care Medicine, “Dr. José E. González” University Hospital, Nuevo León Autonomous University, Av. Francisco I. Madero1500, Suburb: Mitras Centro, Monterrey, NL 64460 Mexico

**Keywords:** Critical care, Eosinophils, Hospital mortality

## Abstract

**Background:**

Predicting mortality in the intensive care unit (ICU) is one of the biggest challenges in critical care medicine. Several studies have linked the presence of eosinopenia with adverse outcomes in different populations.

**Methods:**

We performed a case control study to determine whether the eosinophil count at ICU admission was a predictor of hospital mortality. We included data from patients 18 years or older admitted to the medical or surgical ICU in a university hospital in northern of Mexico. Medical records of 86 non-survivors (cases) and 99 discharged alive patients (controls) were randomly reviewed; clinical records of patients with an ICU stay of less than 24 h and those whose information was incomplete were excluded.

**Results:**

Median of eosinophil count at ICU admission was 0.013 (interquartile range (IQR) 0.00 to 0.57) K/μL. There was no significant statistical difference in eosinophils at admission between survivors and non-survivors (0.014 [IQR 0.00 to 0.36] vs. 0.010 [IQR 0.00 to 0.57] K/μL, *P* = 0.35). In the multivariate analysis, APACHE II score at ICU admission and discharge were the only mortality predictors. Survivors had a significantly greater increase in eosinophil count during the first 7 days of ICU stay (0.104 [IQR −0.64 to 0.41] vs. 0.005 [IQR −1.79 to 0.43] K/μL, *P* = 0.004).

**Conclusions:**

In our study, eosinophil count at ICU admission was not associated with increased hospital mortality. The larger increase in number of eosinophils observed during the first week of ICU stay in surviving patients deserves to be investigated further.

## Background

Predicting mortality in the intensive care unit (ICU) is one of the biggest challenges in critical care medicine. A timely prognosis prediction is essential for proper decision-making. There are several scales to quantify severity of critical illness; most of them were validated in different populations, frequently in the context of clinical trials [1]. Although there is continuous progress in prediction model performance, their use in the individual patient should be considered with caution given its poor sensitivity [2, 3]. In order to achieve a better estimate of mortality, scales based on biomarkers related to presence of oxidative stress or systemic inflammatory response have been developed; among the biomarkers most used in the ICU are C-reactive protein, glucose, and lactate [4–7]. Several studies have linked the presence of eosinopenia with increased risk of death in patients with bacteremia [8], chronic obstructive pulmonary disease exacerbation [9], and in diverse populations admitted to the ICU [10–12]. The use of eosinopenia as a prognostic factor at ICU admission is attractive due to its availability, low cost, and minimum delay between taking blood samples and obtaining results. Its ease of application contrasts with complexity of various scales and algorithms currently used. While the scales with more clinical variables tend to show better performance compared to the simple ones [13], different biomarkers have shown robust prognostic power even when they are used individually [5, 6, 14–16]. The primary aim of this study was to determine whether the eosinophil count at ICU admission is a hospital mortality predictor.

## Methods

### Patients and context

We conducted a case control study using data from patients admitted to medical or surgical ICU from January to September 2013. Our hospital is a teaching tertiary center in northern Mexico; it has 23 intensive care beds: 13 for postoperative or trauma patients and 10 for patients with medical conditions. The study was approved by the ethics committee of Dr. José E. González University Hospital (registration number 14-004 NM).

### Data collection

Medical records of non-survivors (cases) and discharged alive patients (controls) were randomly reviewed. All patients were 18 years or older. For both groups, we collected basic sociodemographic variables, diagnosis at ICU admission, daily eosinophil count during ICU stay, Acute Physiology and Chronic Health Evaluation II (APACHE II) score, Sequential Organ Failure Assessment (SOFA) scale and in-hospital death. For patients with more than one ICU admission or discharge, only the first event was included. We excluded patients who had incomplete clinical records or who were discharged to the general ward, transferred to another hospital or died within the first 24 h since ICU admission. Blood sampling was performed by the nursing staff, blood was collected through the proximal port of a central venous catheter and placed in tubes with ethylenediaminetetraacetic acid; the samples were processed in a CELL-DYN Ruby System (Abbott Diagnostics, Abbott Park, IL) in a central laboratory.

### Statistical analysis

We estimated the sample size based in variable “eosinophil count at ICU admission” with the power.*t*.test function of the R commander (R program, version 2.1.2). We calculated the standard deviation and corresponding mean for survivors and non-survivors to evaluate difference extent and get the size of the effect. The number needed to obtain a power of 80 % and 95 % confidence intervals was 77 patients in each group. We tested normal distribution with the Kolmogorov-Smirnov test. Data are shown as means and standard deviations for variables with normal distribution and as medians and interquartile ranges for non-normal variables. We used the *t* test, the Mann–Whitney *U*-test, ANOVA or chi-square as indicated. We compared eosinophil count at admission between survivors and non-survivors and also compared eosinophil count at discharge and at third and seventh day in patients who remained in ICU at those times. We calculated the receiver operating characteristic (ROC) curve, area under the curve (AUC) and Youden index for eosinophil at ICU admission and compared it with AUC for APACHE II and SOFA. According with sample size, we tested the eight most significant variables in a multivariate analysis by the Cox proportional regression model; the multicollinearity between predictor variables was ruled out by introducing at multivariate analysis only those with Pearson correlation coefficient less than .80. We defined a statistically significant difference as a *P* value less than .05. Analysis was performed using SPSS version 18.0 for Windows (SPSS Inc., Chicago, IL).

## Results

During the study period, 735 patients were admitted at medical or surgical ICU and 179 (24.3 %) died. We included data of 185 patients, 99 (53.3 %) in the survivors group and 86 (46.5 %) in the non-survivors group. Clinical and demographic characteristics are shown in Table 1. Mean age was 47 ± 18 years. Male sex was predominant. The most common cause of ICU admission was a medical disease (47 %). Eighty-six patients had diagnostic criteria for sepsis, 27 (27.3 %) in the survivors and 59 (68.6 %) in the non-survivors group (*P* < .001). Time and cause of death are shown in Table 2. Main mortality cause was septic shock in 53.5 % of patients. Fifty-six (65.1 %) patients died from the disease that caused their admission.

**Table 1:** Clinical and demographic variables at ICU admission

Variable	Total	Survivors	Non-survivors	*P* value
Patients	185 (100.0)	99 (53.5)	86 (46.5)	
Age, years	47 (18.2)	43 (17.4)	51 (18.3)	0.003
Men	128 (69.2)	74 (74.7)	54 (62.8)	0.079
APACHE II, score	16 (1–37)	12 (1–30)	21 (9–37)	<0.001
SOFA, score	6 (0–20)	5 (0–14)	9 (2–20)	<0.001
Predicted mortality, %	25 (4–85)	15 (4–75)	40 (9–85)	<0.001
Comorbidities				
DM2	58 (31.4)	23 (23.2)	35 (40.7)	0.011
Ischemic cardiopathy	24 (13.0)	12 (12.1)	12 (14.0)	0.711
Chronic nephropathy	23 (12.4)	5 (5.1)	18 (20.9)	0.001
Chronic liver disease	7 (3.8)	2 (2.0)	5 (5.8)	0.265
Chronic pneumopathy	7 (3.8)	4 (4.0)	3 (3.5)	1.000
Blood neoplasia	1 (0.5)	1 (1.0)	0 (0.0)	1.000
Solid neoplasia	15 (8.1)	4 (4.4)	11 (12.8)	0.030
HIV infection	5 (2.7)	1 (1.0)	4 (4.7)	0.285
ICU admission diagnosis				
Medical	87 (47.0)	37 (37.4)	50 (58.1)	0.005
Surgical, urgent	40 (21.6)	19 (19.2)	21 (24.4)	0.389
Surgical, elective	25 (13.5)	21 (21.2)	4 (4.7)	0.001
Trauma	33 (17.8)	22 (22.2)	11 (12.8)	0.095
ICU admission eosinophils count, K/μL	0.013 (0.0–0.578)	0.014 (0.0–0.363)	0.010 (0.0–0.578)	0.355
Hospital stay, days	14 (2–96)	18 (3–96)	11.5 (2–56)	0.007
ICU stay, days	6 (1–46)	5 (1–28)	7.5 (1–46)	0.004

Data are shown as number and percentage, mean and standard deviation or median and interquartile range as needed

*ICU* intensive care unit, *APACHE II* acute physiology and chronic health evaluation II, *SOFA* sequential organ failure assessment score, *DM2* type 2 diabetes mellitus, *HIV* human immunodeficiency virus

**Table 2:** Time and death cause in non-survivors group

Cause of death	Day 1 to 7	Day 8 to 14	Day 15 to 21	Day 22 to 28	Day 28 or later	Total
Cancer	1					1
Cardiogenic shock	2	2			1	5
Hypovolemic shock	2					2
Neurologic	13					13
Pulmonary embolism	1					1
Respiratory insufficiency	5	5	1	2	3	16
Septic shock	17	15	7	4	5	48
Total, *n* (%)	41 (47.7)	22 (25.6)	8 (9.3)	6 (7.0)	9 (10.5)	86 (100.0)

Death causes are shown as number; total data are shown as number and percentage

Eosinophil count median at ICU admission was 0.013 (interquartile range (IQR) 0.00 to 0.57) K/μL. There was no statistically significant difference in admission eosinophils between survivor and non-survivor patients (0.014 [IQR 0.00 to 0.36] vs. 0.010 [IQR 0.00 to 0.57] K/μL, *P* = 0.35). In the 86 patients with sepsis, eosinophil count at admission was not different between survivors and non-survivors (0.013 [IQR 0.00 to 0.05] vs. 0.016 [IQR 0.00 to 0.06] K/μL, *P* = 0.44). Taking as a cutoff point, the traditional level of 0.40 K/μL, 126 (67.6 %) patients presented eosinopenia at ICU admission, difference between groups was not significant (64 [64.6 %] vs. 61 [70.9 %], *P* = 0.36).

In univariate analysis the following factors were associated with hospital mortality: age, APACHE II and SOFA at ICU admission and discharge, sepsis, eosinophil count at ICU discharge, type 2 diabetes mellitus, chronic kidney disease, solid neoplasia, a medical diagnosis as admission cause, and discharge to the general ward during a night shift. Eosinophil count at 72 h showed borderline significance (0.13 [IQR 0.0 to 0.90] vs. 0.040 [0.0 to 0.76] K/μL, *P* = .05). ICU stay was 2.5 days longer in the group of non-survivors (5 [IQR 1 to 28] vs. 7.5 [IQR 1 to 46] days, *P* = 0.004). The total hospital stay was shorter in non-survivor group (18 [IQR 3 to 96] vs. 11.5 [2 to 56] days, *P* = 0.007). Patients with elective surgery had a lower mortality (21 [21.2 %] vs. 4 [4.7 %], *P* = 0.001). Seventy-four (74.7 %) survivors and 45 (52.3 %) non-survivor patients remained in ICU for a week or more; those who survived had a significantly greater increase in eosinophil count during the first 7 days of ICU stay (0.104 [IQR −0.64 to 0.41] vs. 0.005 [IQR −1.79 to 0.43] K/μL, *P* = 0.004).

The AUC for eosinophil count at admission, APACHE II and SOFA was 0.53 (IQR 0.45 to 0.62), 0.83 (IQR 0.77 to 0.89), and 0.78 (IQR 0.71 to 0.84), respectively.

The results of the multivariate analysis are shown in Table 3. Only APACHE II score at admission and at discharge significantly predicted hospital mortality.

**Table 3:** Factors associated with hospital mortality, multivariate analysis

Variable	HR	95 % CI	*P* value
Age	0.994	0.97–1.00	0.407
APACHE II at admission	1.039	1.00–1.07	0.048
APACHE II at ICU discharge	1.097	1.06–1.13	<0.001
Hospital stay	0.805	0.75–0.85	<0.001
Eosinophils <0.10 K/μL at 72 h	1.333	0.76–2.32	0.312
Eosinophils <0.03 K/μL at ICU discharge	0.965	0.54–1.70	0.903
Discharge from ICU during night shift	1.165	0.66–2.03	0.591
Medical diagnosis at ICU admission	1.342	0.80–2.23	0.259

*HR* hazard ratio, *CI* confidence interval, *APACHE II* acute physiology and chronic health evaluation II, *SOFA* sequential organ failure assessment score, *ICU* intensive care unit, *DM2* type 2 diabetes mellitus

## Discussion

Eosinophils are pleiotropic, multifunctional cells involved in the initiation and propagation of inflammatory response triggered by diverse stimulus [17]. Their life cycle is tightly regulated by granulocyte colony-stimulating factor, macrophages, IL-3, and IL-5; decrease in their concentration, as occurs during bacterial or fungal sepsis [18], causes eosinophil apoptosis after 48 to 72 h [17, 19]. In 1893, Zappert first described the reduction in eosinophil count related to acute infection [20]. It has been proposed that this decrease is due to at least three mechanisms: 1) peripheral sequestration in inflamed tissue, 2) eosinophils production inhibition, and 3) suppression of mature cell release from the bone marrow [10]. In animal model, there is an up to 80 % reduction in eosinophil count within 6 h after the infective stimulus [21]. Several studies have proposed eosinopenia as a marker for infection [22–28]; in contrast, eosinophilia is infrequent during severe sepsis, and its presence even leads to questioning the infectious etiology of the systemic inflammatory response syndrome [29].

Eosinopenia is frequent and has been linked to mortality in different settings during critical illness; in our study, it was present in 67.5 % of patients, an intermediate value compared to 46.5 % and 86 %, reported by Ho et al. in critically ill patients with bacteremia [7, 28]. We did not find an association between eosinophil count at ICU admission and hospital mortality, this contrast with that reported by other authors [10, 12, 30]. The retrospective design of our study, including an unselected population of critically ill patients and increased frequency of sepsis in the group of non-survivors, could explain this difference. We did not find a difference in eosinophil count at ICU admission between survivor and non-survivor patients with sepsis; the value of this analysis is limited due to a low number of included patients.

In univariate analysis, eosinophil count at 72 h showed borderline significance to predict hospital mortality (0.13 [IQR 0.0 to 0.90] vs. 0.040 [IQR 0.0 to 0.76] K/μL, *P* = 0.05); we build ROC curve and identified 0.103 K/μL as count with a greater discrimination power. However, at multivariate analysis this did not remain as an independent predictor of hospital mortality. Bass et al. found that circulating eosinophil number increases as early as 12 h after appropriate antibiotic treatment initiation [21]. Even though there is no evidence to support the 72 h as a specific point in time at which increase in eosinophil count relates to better outcomes, we arbitrarily defined a 72-h period based on theory that serial measurement of this biomarker could resemble the serial measurement of other markers along evolution of critical illness (e.g., procalcitonin); our results did not support this hypothesis.

A retrospective study of 1446 patients demonstrates that an eosinophil count <0.01 K/μL at ICU discharge is associated with an increased risk of readmission (HR 2.50 [95 % CI 1.38 to 4.50], *P* = 0.002) and hospital mortality (HR 2.65 [95 % CI 1.77 to 3.98], *P* = 0.001) [11]. Our ROC curve analysis placed that point in 0.031 k/μL. Given that up to 10 % of patients discharged from the ICU die before leaving the hospital [31], various scales have been developed to reduce the number of inadequate discharges from the ICU [32–34], but their use is frequently limited due to their complexity. In this regard, eosinopenia is an interesting marker: it may reflect a state of persistent inflammatory response, where the action of various cytokines avoids normalization of eosinophil count. However, in multivariate analysis of our data, eosinophils at ICU discharge were not significant predictors of hospital mortality; lack of significance at multivariate analysis could be due to the low number of patients since the sample size was not calculated for this outcome.

Consistent with the available literature [8, 10, 30], we found that the inability to increase the eosinophil count was a predictor of mortality. In our study, survivors showed a 20-fold increase in the eosinophil count at the seventh day compared to non-survivors. The increase in eosinophil number during the first week of ICU stay in patients who survived is a valuable result given the biological context that relates their presence with the resolution of the inflammatory state [7, 21]. This aspect becomes even more valuable given the easy access and low cost of blood cells count.

Our study has several limitations. First, its retrospective design involved a limited value in the quality of information; in addition, the background of comparative groups was different and potentially included several undetermined confounding factors. Second, we did not take into account the percentage of eosinophils with respect to total leukocyte count; this factor was related to an increase in predictive power, sensitivity, and AUC for mortality prediction in one study [12]. Third, patients were not stratified according to steroids or vasoactive amines use; these substances have been associated with eosinophils apoptosis [19]. Finally, the study was conducted at a single center.

## Conclusions

In this study, the eosinophil count at admission to the ICU was not associated with increased in hospital mortality. In our population, the APACHE II score at ICU admission and discharge remains as the best mortality predictors. The larger increase in eosinophil number during the first 7 days of ICU stay observed in survivor patients is a finding that deserves to be investigated in future studies.

## Abbreviations

ICU: intensive care unit
IQR: interquartile range
APACHE II: Acute Physiology and Chronic Health Evaluation II
SOFA: Sequential Organ Failure Scale
ROC: receiver operating characteristic
AUC: area under curve
HR: hazard ratio
